# Flexible nasolaryngoscopy accuracy in laryngomalacia diagnosis

**DOI:** 10.1016/S1808-8694(15)30747-3

**Published:** 2015-10-19

**Authors:** Tania Mara Assis Lima, Denise Utsch Gonçalves, Lucas V. Gonçalves, Paulo Augusto C. Reis, Angela Beatriz S. Lana, Fernando F. Guimarães

**Affiliations:** 1 PhD in Otolaryngology, Adjunct Professor - Department of Otolaryngology - Medical School - UFMG; 2 PhD. Adjunct Professor - Department of Otolaryngology - Medical School - UFMG; 3 Resident physician in Otolaryngology - University of Minas Gerais Medical School; 4 Resident physician in Otolaryngology - University of Minas Gerais Medical School; 5 Resident physician in Otolaryngology - University of Minas Gerais Medical School‥; 6 Resident physician in Otolaryngology - University of Minas Gerais Medical School‥

**Keywords:** diagnosis, larynx, abnormalities, laryngoscopy

## Abstract

Laryngomalacia is the most common cause of stridor in infancy. Diagnosis is established by associating the clinical manifestations with laryngoscopic findings in a dynamic form.

**Aim:**

To analyze diagnostic accuracy of laryngomalacia through flexible nasolaryngoscopy performed by four different examiners. Form of studying: Comparison of diagnostic tests (clinical study).

**Material and method:**

A protocol of videolaryngoscopic evaluation for patients with laryngomalacia was created encompassing the following items: anterior displacement of the arytenoids; omega-shaped epiglottis; short aryepiglottic folds; posterior displacement of epiglottis; vocal folds being visible or not; edema of the posterior larynx. Four different examiners analyzed the videolaryngoscopic examinations of 18 patients with established diagnosis of laryngomalacia previously established by clinical data, epidemiology and anatomical traits. The four observers knew previously that the patients had laryngomalacia and which criteria should be analyzed in the evaluation protocol. Observers were unaware of the results each one found.

**Results:**

diagnostic agreement average considering all parameters evaluated was of 88.2%.

**Discussion/Conclusion:**

Dynamic flexible nasolaryngoscopy is a proven diagnostic method, regardless of physician experience.

## INTRODUCTION

Laryngomalacia is the most common cause of stridor in children, being the culprit for 65 to 75% of the stridor cases^1^. It affects more men then women, at a 2:1 rate. This condition is established by the laryngeal failure in keeping its lumen open during inhaling. Inspirational stridor is the main disease manifestation^2^ which is usually self limited and resolves in the first 12 to 18 months of life. Nonetheless, in 10% of the cases it is necessary to operate, because the respiratory obstruction can cause severe dyspnea, dysphagia, growth delay and obstructive sleep apnea^1^.

Its etiology is still unknown. Numerous theories try to explain its etiopathogenesis. The one most commonly accepted today states that laryngomalacia results from a neuromuscular immaturity, causing supraglottic hypotonia and bringing laxity to these structures during inhaling^2^. Another theory is associated with a reduction in support from the cartilaginous framework. Another possible etiology is laryngeal muscles hypotonia.

Diagnosis is based on clinical and epidemiological history of newborns and infants with inspiratory stridor, which worsens with restlessness or crying, and improves during sleep. Usually, direct laryngoscopy is the gold standard exam to diagnose stridor^3^, ^4^, ^5^. Since laryngomalacia is the most common cause of stridor and, usually the most benign cause, a dynamic laryngeal assessment by means of a nasolaryngoscopy through flexible endoscope has shown good results, although it does not replace direct laryngoscopy with the rigid endoscope in indicated cases.^6^ However, endoscopic image assessment has not been properly systematized until current days, with many confounding factors associated with examiner's experience and anatomical variation inherent to human beings. Therefore, endoscopic image evaluations have not been systematized enough, with confounding factors associated with the observer's experience and to the anatomical variations inherent to human beings. Thus, we still need to establish, in a controlled fashion, which anatomical alterations prove to be more constantly present when evaluated by nasal-fibroscopy, setting up the parameters that must be evaluated in all cases with suspicion of laryngomalacia. It seems that this has not been done until current times.

The goal of the present investigation is to establish videolaryngoscopy diagnostic accuracy regarding laryngomalacia, when performed by observers with variable clinical experience in laryngology.

## MATERIALS AND METHODS

This is an agreement study, used to assess the reliability of a diagnostic test. The project was evaluated by the Ethics in Research Committee of the Federal University of Minas Gerais, under protocol # 571/07.

Eighteen patients with prior diagnosis of laryngomalacia - based on clinical, epidemiological and anatomical data, were evaluated. The videolaryngoscopic exams of these patients were compared in relation to the agreement index from four different examiners. The videolaryngoscopic assessment protocol used was based on the most frequently observed anatomical alterations found in this disorder2. The parameters considered were:

1) anterior arytenoid drawing during inhaling;
2) tubular epiglottis (omega-shape) which collapses during inhaling;
3) short aryepiglottic folds;
4) posterior epiglottic drawing;
5) good or bad view of the vocal folds;
6) edema on the posterior laryngeal structures. The exams were carried out with a flexible nasal-laryngoscope and recorded on a video-tape.

The examiners had between t (examiners 1, 2 and 3) and 15 years (examiner 4) of clinical experience in children laryngology. They all had prior knowledge that the patients had been diagnosed with laryngomalacia and also knew what the anatomical criteria to be considered were.

The analyses were carried out in such a way that one observer did not know what the others had found. Later on, all the observers analyzed the exams together and reached a consensus, which was then considered reference. Therefore, in order to establish a gold standard, we considered the consensus of the exams and not the total number of patients. We were interested in checking the agreement between the alterations noted by the examiners in relation to the consensus (gold standard). In comparing, each examiner was assessed as to the number of correct findings in relation to the gold standard.

## RESULTS

The analysis encompassing the eighteen laryngoscopies carried out by each examiner and the consensus on the assessment are reported on Table 1. The average agreement of all the parameters considered in relation to the consensus of the four examiners was of 88.2%. The data associated with such analysis is specified on Table 2.

**Table 1:** Parameters considered on the videolaryngoscopic exam performed by four examiners and the consensus of such analysis regarding the diagnosis of 18 patients with laryngomalacia.

Videolaryngoscopic exam parameters	Examiners				Consensus
	1	2	3	4	
Anterior arytenoid drawing	12	14	15	16	16
Omega-shaped epiglottis	11	14	13	11	11
Short aryepiglottic folds	17	18	17	17	18
Posterior epiglottis drawing	1	2	1	1	1
No visualization of vocal folds	5	3	5	4	4
Posterior larynx edema	14	16	13	13	15

**Table 2:** Agreement index from four examiners in relation to the consensus results of 16 videolaryngoscopic exams from patients with laryngomalacia.

Vediolaryngoscopic exam parameters	Examiners' evaluations				Agreement rate
	1	2	3	4	
Anterior arytenoid Drawing	12/16 (75%)	14/16 (87.5%)	15/16 (93%)	16/16 (100%)	88.8%
Omega-shaped epiglottis	11/11 (100%)	7/11 (63%)	9/11 (81%)	11/11 (100%)	86.0%
Short aryepiglottic folds	17/18 (94.4%)	18/18 (100%)	17/18 (94.4%)	17/18 (94.4%)	95.8%
Posterior epiglottic Drawing	1/1 (100%)	0,5/1 (50%)	1/1 (100%)	1/1 (100%)	87.5%
Not possible to see vocal folds	3/4 (75%)	3/4 (75%)	3/4 (75%)	4/4 (100%)	81.4%
Posterior laryngeal edema	14/15 (93.3%)	14/15 (93.3%)	13/15 (86.6%)	13/15 (86.6%)	89.9%

As to the individual analysis, examiner 4, with 15 years of clinical experience, was the one who most disagreed in terms of the “Posterior Laryngeal Edema” parameter, when compared to the other examiners with 2 years of clinical experience (Table 2).

## DISCUSSION

Nasal-laryngoscopy is a very important exam in otorhinolaryngology, and it is increasingly more indicated in the evaluation of upper airway affections. Since it is an examiner-dependent exam, it is subject to perception variations mainly associated with the examiner's clinical experience.

As far as the laryngomalacia diagnosis is concerned, the agreement average for the parameters assessed was of 88.2% among examiners. The most reliable parameter was “short aryepiglottic folds”, of which agreement rate was of 95.8%. The less reliable was “not visualizing the vocal folds”, in which the agreement was of 81.4%. Not being able to see the vocal folds in the videolaryngoscopic evaluation has been associated with the prognosis, because some authors use such information, together with the clinical picture, in order to indicate surgery^7^.

In the present investigation, the agreement rate, considering all the parameters evaluated, was high, proving that the exam is reliable for the diagnosis of laryngomalacia, even when performed by experts with little experience in laryngology.

Two parameters must be stressed:

1) vocal folds visualization or not, because examiners with less experience in laryngology had difficulty in identifying it, and this is one of the parameters considered for surgery indication^7^;
2) edema on the posterior laryngeal structures^8^, was more easily identified by the examiner with greater clinical experience.

## CONCLUSION

Nasal-laryngoscopy is a good exam for the diagnosis of laryngomalacia, bearing a sensitivity of 88.2% - regardless of examiner's clinical experience. The most reliable parameter was the evaluation of short aryepiglottic folds. For cases of possible surgical indication and in order to assess the presence of edema on the posterior laryngeal structures, clinical experience in children laryngology is paramount.
